# Notch1 Protects against Ischemic-Reperfusion Injury by Suppressing PTEN-Pink1-Mediated Mitochondrial Dysfunction and Mitophagy

**DOI:** 10.3390/cells12010137

**Published:** 2022-12-29

**Authors:** Qirong Xu, Sheng Liu, Qiang Gong, Rongrong Zhu, Jichun Liu, Qicai Wu, Xueliang Zhou

**Affiliations:** 1 Department of Thoracic Surgery, The First Affiliated Hospital, Nanchang University, Nanchang 330006, China; ndyfy02370@ncu.edu.cn; 2 Department of Cardiac Surgery, The First Affiliated Hospital, Nanchang University, Nanchang 330006, China; ndyfy01290@ncu.edu.cn (S.L.); gongqiang@ncu.edu.cn (Q.G.); ndefy18002@ncu.edu.cn (J.L.); 3 Department of Cardiology, Jiangxi Hospital of Traditional Chinese Medicine, Jiangxi University of Chinese Medicine, Nanchang 330006, China; zhurongrong@jxutcm.edu.cn

**Keywords:** Notch1, PTEN, Pink1-Mfn2-Parkin, ischemic-reperfusion injury

## Abstract

Background: Myocardial ischemia/reperfusion injury is associated with adverse cardiovascular outcomes after acute myocardial infarction. However, the molecular mechanism of ischemia/reperfusion injury remains unclear. Mitochondria dysfunction is a participant in and regulator of myocardial ischemia-reperfusion injury. However, the molecular mechanisms involved in this process are not yet fully understood. We previously reported that Notch1 can reduce mitochondrial lysis, reduce myocardial infarct size, and inhibit ventricular remodeling. Herein, we explore the role of the downstream target Notch1 in mitochondrial regulation. Methods: This study constructs an ischemic/reperfusion injury rat model and a hypoxia/reoxygenation cell model. The expression of PTEN is detected by real-time PCR, Western blot, and immunofluorescence staining. Cell viability is analyzed with CCK-8. Apoptosis level is detected via the TUNEL assay, and mitochondrial fission/fusion is analyzed with MitoTracker Green staining. Cardiac troponin I (cTnI), lactate dehydrogenase (LDH), superoxide dismutase (SOD), and CK levels of creatine kinase-MB (CK) are measured with ELISA kits. Results: We found that PETN-Pink1-Parkin signaling is inhibited by Notch1 I/R in injured neonatal cardiomyocytes and hearts, i.e., via the inhibition of mitochondrial dysfunction and fragmentation. With the recure of PTEN or Pink1, the protective effect of Notch1 was largely diminished. Conclusion: These results suggest that N1ICD acts protectively against ischemic reperfusion injury by suppressing PTEN-Pink1-mediated mitochondrial dysfunction and fragmentation.

## 1. Background

Reperfusion is the only way to salvage ischemic myocardial damage from infarction, but reperfusion itself can cause additional damage, so the resulting myocardial infarction size is determined by both ischemia and reperfusion injury. Myocardial ischemia/reperfusion injury (I/R) is a major health problem in China, and its morbidity and mortality are increasing every year [1,2,3,4]. The development of acute heart failure is the leading cause of death after acute myocardial ischemia/reperfusion injury. Advances in medical technology, such as medical thrombolysis, interventional stents, and bypass surgery, have significantly reduced mortality in patients with acute myocardial I/R and thus reduced the experience of acute heart failure [4,5,6,7,8,9,10]. Promoting myocardial I/R region repair is critical to preventing heart failure and reducing long-term mortality after I/R.

The neurogenic locus notch homolog protein (Notch) signaling pathway plays a role in regulating cardiac development and the proliferation and differentiation of cardiomyocytes [11]. The Notch1 intracytoplasmic domain (N1ICD) translocates into the nucleus and regulates Hes1 gene expression by binding to ubiquitous transcription factors and centromere-binding protein 1. Studies have shown that the Notch signaling pathway plays an important role in the genesis, development, and pathophysiology of the cardiovascular system [12]. The role of the Notch1 receptor and its downstream signaling molecule Hes1 in ischemic heart disease has been studied [12]. The activation of Notch1 during myocardial I/R may activate downstream Hes1 molecules, inhibiting the expression of phosphatase and tensin homologs and mitigating myocardial I/R damage. The activation of the myocardial Notch1 signaling pathway protects the ischemic myocardium by activating downstream Hes1 signaling and alleviating MI/R injury-induced oxidative stress and nitrate stress injury [13]. The Akt signaling pathway plays a key role in cell survival, growth, and migration. It may also be a survival signal during myocardial ischemia, and activation of this signal can exert an anti-apoptotic effect through both upstream and downstream signaling pathways [13,14,15,16]. We previously reported that Notch1 regulates the dynamic balance between mitochondrial fusion/division and mitochondrial autophagy in I/R injury cardiomyocytes and provides protection for cardiomyocytes [17,18]. It is reported that Notch1 is associated with the regulation of PTEN through the recruitment of C-promoter-binding factor-1 (CBF-1), a Notch transcription factor, and binding to the PTEN promoter [19]. On the other hand, the PTEN-Pink1-MFN2-Parkin signaling pathway is largely involved in the progress of myocardial ischemia/reperfusion injury [20]. However, the regulatory mechanism of the Notch1 signaling pathway on the PTEN-Pink1-MFN2-Parkin signaling pathway and its actual role in I/R injury are still unclear.

Herein, we demonstrate that Notch1 impairs the elevated expression of PETN in I/R heart injuries and cardiomyocytes. Moreover, we found that PTEN over-expression impaired the protective effect of N1ICD against hypoxia/reoxygenation-induced injury, mitochondrial fragmentation, and mitophagy in neonatal cardiomyocytes and hearts. These results suggest that Notch1-regulated PTEN-Pink1-MFN2-Parkin signaling could become a promising therapeutic strategy for treating myocardial I/R injury.

## 2. Methods

### 2.1. Animal Care

The animals used in this study were maintained following the Guide for the Care and Use of Laboratory Animals (Publication 85-23, National Institutes of Health, Bethesda, MD, USA), and all procedures were approved by the First Affiliated Hospital of Nanchang University (Nanchang, Jiangxi, China).

### 2.2. Isolation and Culture of Rat Neonatal Ventricular Myocytes

Rat neonatal left ventricular myocytes were isolated from neonatal rat hearts using a standard enzymatic method as previously described [18].

### 2.3. Simulated H/R in Isolated Cardiomyocytes

A cellular model of simulated H/R (20 min/30 min) in neonatal left ventricular myocytes was used as previously described [21]. Briefly, the primary neonatal left ventricular myocytes were cultured in an anoxic solution in an anoxic incubator (95% N_2_/5% CO_2_) for 3 h. The hypoxia solution was then replaced by a reoxygenation solution and cultured in a high-oxygen incubator (95% O_2_/5%-CO_2_) for 3 h.

### 2.4. Construction and Infection of Recombinant Adenoviruses

Recombinant adenoviruses expressing rat N1ICD/PTEN/Pink1 complementary DNA (cDNA) were prepared using the pAdEasyTM vector system (Qiagen, Venlo, The Netherlands) as described previously. Primary cardiac myocytes were infected with adenoviral particles with a multiplicity of infection of 100.

### 2.5. Cell Viability Assay

The cell viability of neonatal cardiomyocytes was detected with CCK-8 assay (Dojindo) as described previously [21].

### 2.6. Real-Time PCR

Total RNA was extracted from frozen heart tissues or cultured cells, and RNA reverse-transcription was performed as previously described. RT-PCR was conducted using an SYBR Green Master Mix (Cowin, Beijing, China). All experiments were independently repeated three times.

### 2.7. Western Blot Analysis

The adult cardiomyocytes were lysed in cell lysis buffer (Beyotime Institute of Biotechnology, Nantong, China) at 4 °C. Protein samples were separated by 8–10% SDS-PAGE and then transferred to nitrocellulose membranes (Millipore). Membranes were incubated with primary antibodies overnight at 4 °C, followed by incubation with secondary antibodies at room temperature for 1 h. The fluorescent signals were detected using enhanced chemiluminescence via ImageQuant LAS4000 (GE). All experiments were independently repeated three times.

### 2.8. Immunofluorescence Analysis

Immunofluorescence assay was carried out as described above. The muscle cells fixed with paraformaldehyde were treated with 0.5% Triton X-100 PBS for 15 min. The myocytes were then immunostained with an anti-PTEN (1:100) antibody. After washing with PBS, FITC-conjugated anti-rabbit IgG samples (1:2000, Jackson, MS, USA) were stained for 2 h. The stained cells were observed under a Zeiss LSM800 confocal microscope (Zeiss, Heidelberg, Germany).

### 2.9. Hes1 and PTEN Promoter-Luciferase Reporter Assay

A commercial PTEN luciferase reporter kit from SABiosciences Qiagen was used to determine the effect of HES1 over-expression on the transcriptional activity of PTEN. Neonatal cardiomyocytes cells were transfected with the reporter construct using Lipofectamine 3000 (Thermo Fisher, Waltham, MA, USA). Twenty-four hours after transfection, cells were harvested using lysis buffer. Samples were centrifuged, and a 20 μL aliquot was used for the measurement of dual-luciferase activity using a luminometer.

### 2.10. TUNEL Staining

Apoptosis rates in cultured neonatal cardiomyocytes and mouse heart tissue sections were analyzed via TUNEL staining using the in situ cell death detection kit (Roche Applied Science, Upper Bavaria, Germany) according to the manufacturer’s protocol. In summary, the slides were incubated with the TUNEL reaction mixture, apoptotic cells were labeled, and the total number of cells was determined by DAPI staining. The slides were viewed under a Zeiss LSM800 laser scanning confocal microscope (Zeiss, Wetzlar, Germany). The apoptosis rate was calculated as the percentage of TUNEL-positive cells in the total number of DAPI-stained cells. All experiments were independently repeated three times.

### 2.11. Measurement of cTnI, Lactate Dehydrogenase (LDH), Superoxide Dismutase (SOD), and CK Levels of Creatine Kinase-MB (CK)

The coronary effluent and culture medium of the cardiomyocytes after reperfusion were placed in a thermostatic chamber. An electrochemiluminescence immunoassay was used to detect the level of cTnI, LDH, and CK according to the instructions of the kit (Roche, Germany). The measurement of SOD activity was performed according to the test kit instructions (Nanjing Kaiji Bio, Nanjing, China). All experiments were independently repeated three times.

### 2.12. Seahorse Analysis

As previously described, mitochondrial metabolic flux was tested in adult cardiomyocytes. Briefly, cardiomyocytes were inoculated in a XF24 hippocampal plate coated with laminin at a density of 104 cells per well and cultured overnight in BCAA-free substrate-restricted medium, then analyzed. OCR and ECAR were determined using the seahorse XF24 extracellular flux analyzer (Seahorse Bioscience, North Billerica, MA, USA). All experiments were independently repeated three times.

### 2.13. Mitochondrial Fusion/Fission Detection

The mitochondrial fusion/fission detection of myocardiocytes was detected as described previously [22].

### 2.14. I/R Injury Model of Langendorff-Perfused Rat Hearts

The I/R injury model in Langendorff-perfused rat hearts was performed as previously described [22]. In brief, the rats were anesthetized with sodium pentobarbital (45 mg/kg I.P.), the hearts were rapidly resected, and the Krebs–Henseleit (K-H) solution was infused at 37 °C using a Langendorff apparatus at a constant pressure of 80 mm Hg as described in the previous paper. A water-filled latex balloon was connected to a pressure sensor (Gould P23DB, AD instrument) and inserted into the left ventricular cavity to achieve a stable LVEDP of 5–10 mmHg during the initial equilibrium. After balanced perfusion, the heart was ischemic for 30 min, followed by another 45 min of perfusion. LVDP and ±dp/dt max were evaluated using the PowerLab system (AD instrument, Dunedin, New Zealand).

### 2.15. In Vivo Adenoviral Gene Delivery

The surgical procedures and adenoviral delivery were carried out as described [22].

### 2.16. Statistical Analysis

Data are expressed as mean ± SEM. Statistical significance was determined by multiple comparisons or repeated measures using analysis of variance or repeated analysis of variance. The Student’s *t*-test was used to estimate the significant difference between the two averages. *p* < 0.05 was considered statistically significant.

## 3. Results

### 3.1. N1ICD Impaired the Hypoxia/Reoxygenation-Elevated Expression of PETN in Neonatal Cardiomyocytes

**Figure 1:**
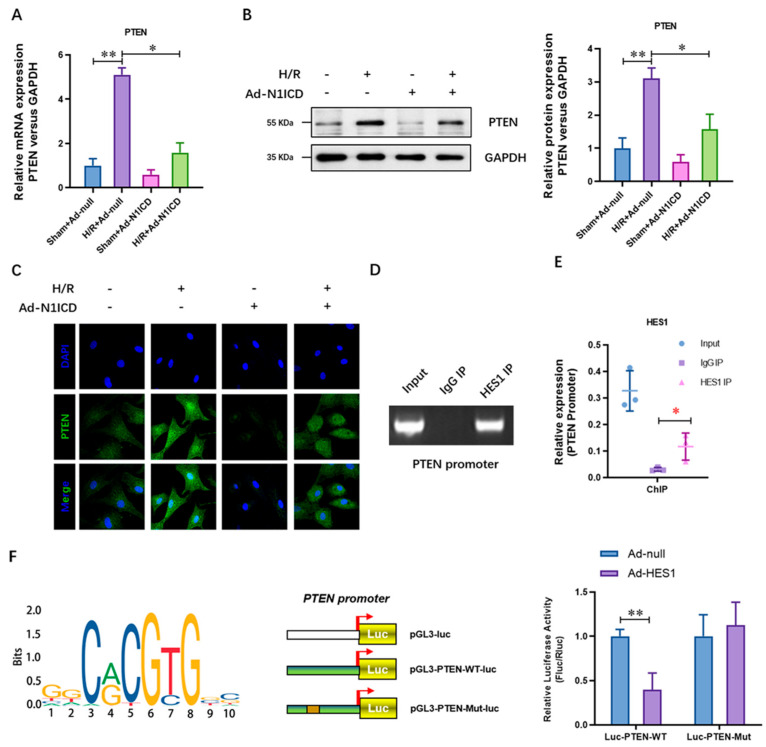
N1ICD impaired the hypoxia/reoxygenation elevated expression of PTEN in neonatal cardiomyocytes. (**A**) The mRNA level of PTEN was analyzed by real-time PCR; (**B**) the protein level of PTEN was analyzed by real-time PCR; (**C**) the protein level of PTEN was analyzed by immunofluorescence staining assay; (**D**) ChIP-RT-PCR and (**E**) ChIP-qPCR were performed to analyze the enrichment of PTEN promoter in the ChIP products of Hes1; (**F**) the promoter activity of PTEN was evaluated by luciferase assay; N = 3; * *p* < 0.05, ** *p* < 0.01 versus indicated group.

To illustrate the effect of N1ICD on the expression level of PTEN in H/R-injured neonatal cardiomyocytes, we overexpressed N1ICD in the sham or H/R-injured neonatal cardiomyocytes with adenovirus. The levels of PTEN mRNA (Figure 1A) and protein (Figure 1B) were significantly increased in the H/R group, whereas N1ICD over-expression dramatically decreased. This decrease was further confirmed by immunofluorescence staining (Figure 1C). Considering Hes1 is a critical transcript factor of Notch1 signaling, we then tested whether Hes1 transcriptionally regulates the expression of PTEN. RT-PCR (Figure 1D), and the real-time PCR (Figure 1E) of the chromatin immunoprecipitation (ChIP) assay showed that the promoters of PTEN were obviously enriched in the components of the HES1 IP products. We then predicted the binding site of Hes1 for the promoter of PTEN with the JASPAR tool (http://jaspar.genereg.net/ accessed on 15 October 2022) and constructed a luciferase promoter activity reporter with wild-type PTEN or mutant promoter sequences (Figure 1D). The luciferase result showed that the transcriptional activity of PTEN was inhibited by Hes1 over-expression (Figure 1F). Moreover, the dual-luciferase assay of the activity of the PTEN promoter suggested that Hes1 significantly decreased the activity of the wild-type PTEN promoter but not the binding site mutant NRF2 promoter (Figure 1F). These results demonstrate that N1ICD impaired the hypoxia/reoxygenation-elevated expression of PETN in neonatal cardiomyocytes.

### 3.2. PTEN Over-Expression Impaired the Protective Effect of N1ICD against Hypoxia/Reoxygenation-Induced Injury in Neonatal Cardiomyocytes

**Figure 2:**
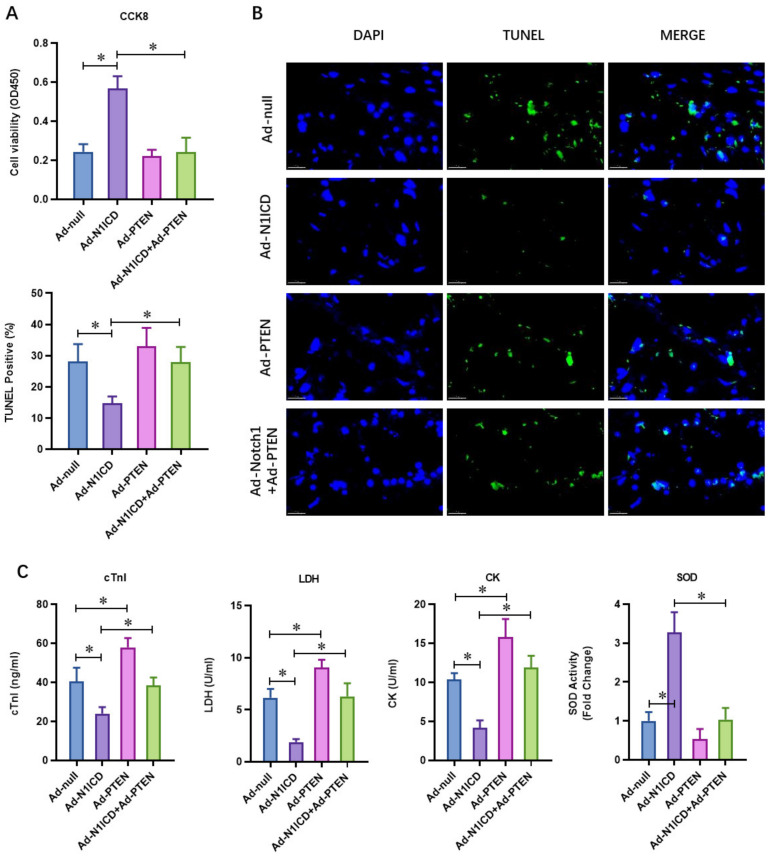
PTEN over-expression impaired the protective effect of N1ICD against hypoxia/reoxygenation-induced injury in neonatal cardiomyocytes. (**A**) The cellular viability was detected by CCK-8 assay; (**B**) the percentage of apoptotic neonatal cardiomyocytes was analyzed by TUNEL staining; (**C**) the level of cTnI, LDH, CK and SOD in the culture medium was also detected by ELISA kits at the end of the reperfusion to assess myocardial injury; N = 3; * *p* < 0.05 versus indicated group.

To explore whether the N1ICD-decreased PTEN expression contributed to the cardioprotection effect of N1ICD, we overexpressed N1ICD with or without PTEN in the H/R-injured neonatal cardiomyocytes with adenovirus. Notch1 improved the cell viability of the H/R-injured neonatal cardiomyocytes (Figure 2A). PETN did not affect the cell viability; however, cell viability was obviously decreased in Notch1-over-expressed H/R neonatal cardiomyocytes (Figure 2A). Considering that apoptosis is the major pathophysiological process underlying liver ischemia/reperfusion (I/R) injury, we analyzed H/R-induced apoptosis via TUNEL staining. The percentage of H/R-induced apoptotic cardiomyocytes decreased with Notch1 over-expression (Figure 2B), which was dramatically elevated by PTEN over-expression. As cTnI is 100% tissue-specific for myocardial damage and is an excellent marker for myocardial injury, cardiac troponin I (cTnI) levels in the culture medium were also detected at the end of the reperfusion to assess myocardial injury. The results showed that the Notch1-over-expression-impaired cTnI level was dramatically elevated by PTEN over-expression (Figure 2C). Consistently, the activity of LDH (Figure 2C) and CK (Figure 2C), which were also used as indicators of myocardial injury, were markedly decreased following H/R in the Notch1-over-expression group. However, PTEN over-expression markedly reversed these effects induced by H/R. Concurrently, the activity of the antioxidative cytokine SOD was evaluated in the Notch1-over-expression group but not in the PTEN-over-expression group (Figure 2C). These results demonstrate that PTEN over-expression impairs the protective effect of N1ICD against hypoxia/reoxygenation-induced injury in neonatal cardiomyocytes.

### 3.3. PTEN Over-Expression Impairs the Protective Effect of N1ICD against Hypoxia/Reoxygenation-Induced Mitochondrial Dysfunction and Fragmentation in Neonatal Cardiomyocytes

**Figure 3:**
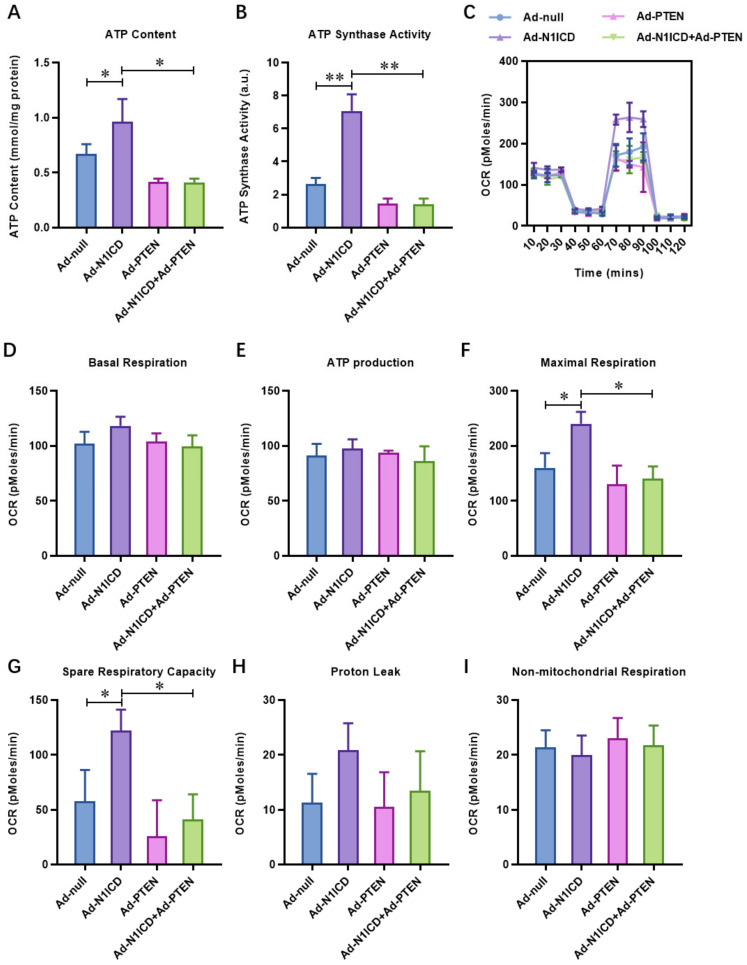
PTEN over-expression impaired the protective effect of N1ICD against hypoxia/reoxygenation induced mitochondrial dysfunction in neonatal cardiomyocytes. (**A**) The ATP content and (**B**) ATP synthase activity was analyzed by quantitative kits; (**C**) the mitochondrial bioenergetics were measured by the seahorse XFp Extracellular Flux Analyzer. The (**D**) basal respiration (BR), (**E**) ATP production, (**F**) maximal respiration (MR), and (**G**) spare respiratory capacity (SPR), (**H**) proton leak, and (**I**) non-mitochondrial respiration were analyzed; N = 3; * *p* < 0.05, ** *p* < 0.01 versus indicated group.

**Figure 4:**
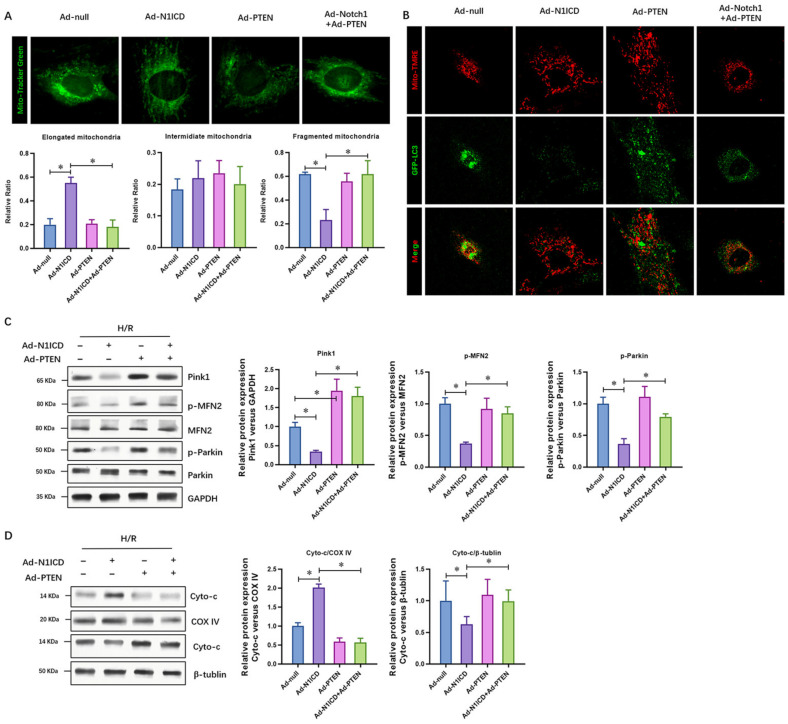
PTEN over-expression impaired the protective effect of N1ICD against hypoxia/reoxygenation-induced mitochondrial fragmentation and mitophagy in neonatal cardiomyocytes. (**A**) Mito-tracker Green staining and confocal microscopy were used to observe the morphology of mitochondrial fragmentation in neonatal cardiomyocytes cells; (**B**) the mitophagy in cardiomyocytes cells was analyzed via the colocalization of GFP-LC3 puncta with Mito-TMRE-stained mitochondria as an index of mitophagy in natal cardiomyocytes cells; (**C**) the regulatory molecules of mitochondrial fission and fusion, including Pink1, Parkin1, and MFN2, were analyzed; (**D**) the regulatory molecule of mitochondrial pathway apoptosis, indicated by cytochrome c release from mitochondria, was analyzed; N = 3; * *p* < 0.05 versus indicated group.

To further verify the Notch1-protected mitochondrial respiration in H/R-injured neonatal cardiomyocytes via the suppression of PTEN, we analyzed the mitochondrial function and respiration of neonatal cardiomyocytes with Notch1 with or without PTEN over-expression. We found that the ATP content (Figure 3A) and ATP synthase activity (Figure 3B) were significantly increased by Notch1 over-expression. PTEN over-expression dramatically impaired the ATP content (Figure 3A) and ATP synthase activity (Figure 3B). Furthermore, the oxygen consumption rate of the neonatal cardiomyocytes, including maximal respiration (MR) and spare respiratory capacity (SPR), increased with Notch1 over-expression and decreased with PTEN over-expression (Figure 3C). Consistently, the H/R-induced mitochondrial fragmentation indicated by the fragmented mitochondrial numbers also decreased with Notch1 over-expression, which was reversed by PTEN over-expression (Figure 4A). Furthermore, the level of mitochondrial fragmentation markers, including Pink1, phosphorylated MFN2, and phosphorylated Parkin, were inhibited by Notch1 over-expression but reversed by PTEN over-expression (Figure 4B). Mitophagy, a process that selectively removes damaged organelles via autolysosome degradation, is an early cellular response to ischemia in cardiomyocytes during I/R and cardiac ischemic diseases. In our results, Notch1 obviously inhibited the H/R-induced mitophagy in neonatal cardiomyocytes (Figure 4C). Mitochondrial fragmentation in apoptosis mitochondrial outer-membrane permeabilization is associated with pro-apoptotic stimuli, which results in the release of cytochrome c from the mitochondria. We also found that Notch1 over-expression significantly decreased the cytoplastic cytochrome c, whereas PTEN over-expression elevated it (Figure 4D). Collectively, PTEN over-expression impaired the protective effect of N1ICD against hypoxia/reoxygenation-induced mitochondrial dysfunction and fragmentation in neonatal cardiomyocytes.

### 3.4. Pink1 Over-Expression Impaired the Protective Effect of N1ICD against Hypoxia/Reoxygenation-Induced Injury in Neonatal Cardiomyocytes

**Figure 5:**
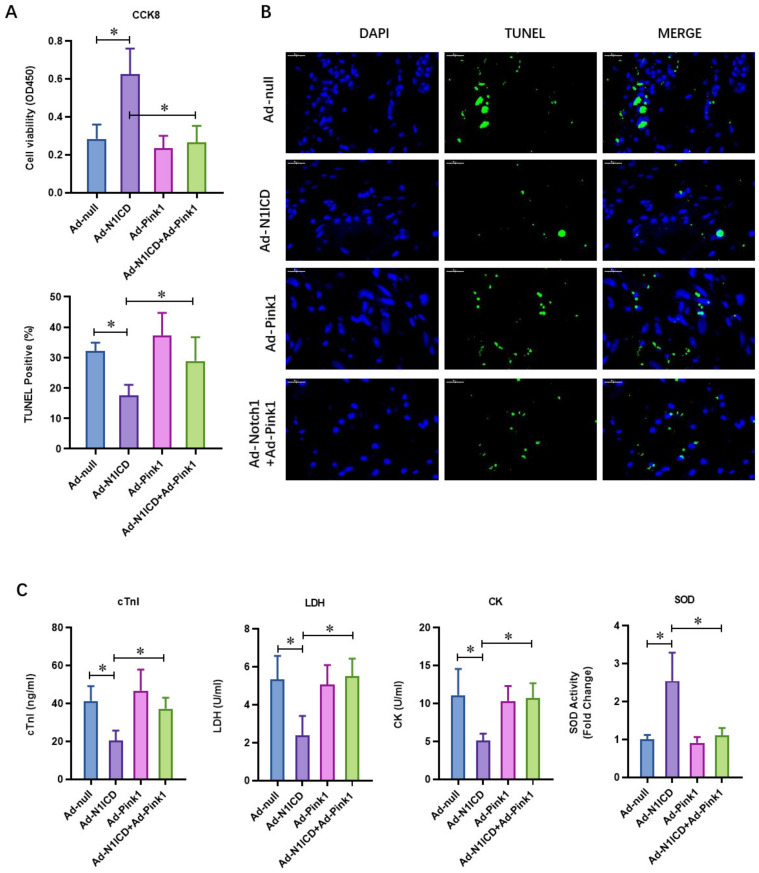
Pink1 over-expression impaired the protective effect of N1ICD against hypoxia/reoxygenation-induced injury in neonatal cardiomyocytes. (**A**) The cellular viability was detected by CCK-8 assay; (**B**) the percentage of apoptotic neonatal cardiomyocytes was analyzed by TUNEL staining; (**C**) the level of cTnI, LDH, CK, and SOD in the culture medium was also detected by ELISA kits at the end of the reperfusion to assess myocardial injury; N = 3; * *p* < 0.05 versus indicated group.

To verify whether Pink1 signaling is critical to the PTEN-impaired cardio protection effect of Notch1, we overexpressed N1ICD with or without Pink1 over-expression in the H/R-injured neonatal cardiomyocytes with adenovirus. Similarly, Notch1 improved the cell viability of H/R-injured neonatal cardiomyocytes (Figure 5A), whereas Pink1 did not affect the cell viability. Moreover, Pink1 over-expression obviously decreased the cell viability in Notch1-over-expressed H/R neonatal cardiomyocytes (Figure 5A). The percentage of Notch1-decreased apoptosis in H/R neonatal cardiomyocytes was increased by Pink1 over-expression (Figure 5B). Moreover, the Notch1-decreased markers for myocardial injury, including cTnI, LDH activity (Figure 5C), and CK activity (Figure 5C), were markedly increased following H/R in the Pink1-over-expression group. These results demonstrate that Pink1 over-expression impairs the protective effect of N1ICD against hypoxia/reoxygenation-induced injury in neonatal cardiomyocytes.

### 3.5. Pink1 Over-Expression Impairs the Protective Effect of N1ICD against Hypoxia/Reoxygenation-Induced Mitochondrial Dysfunction and Fragmentation in Neonatal Cardiomyocytes

**Figure 6:**
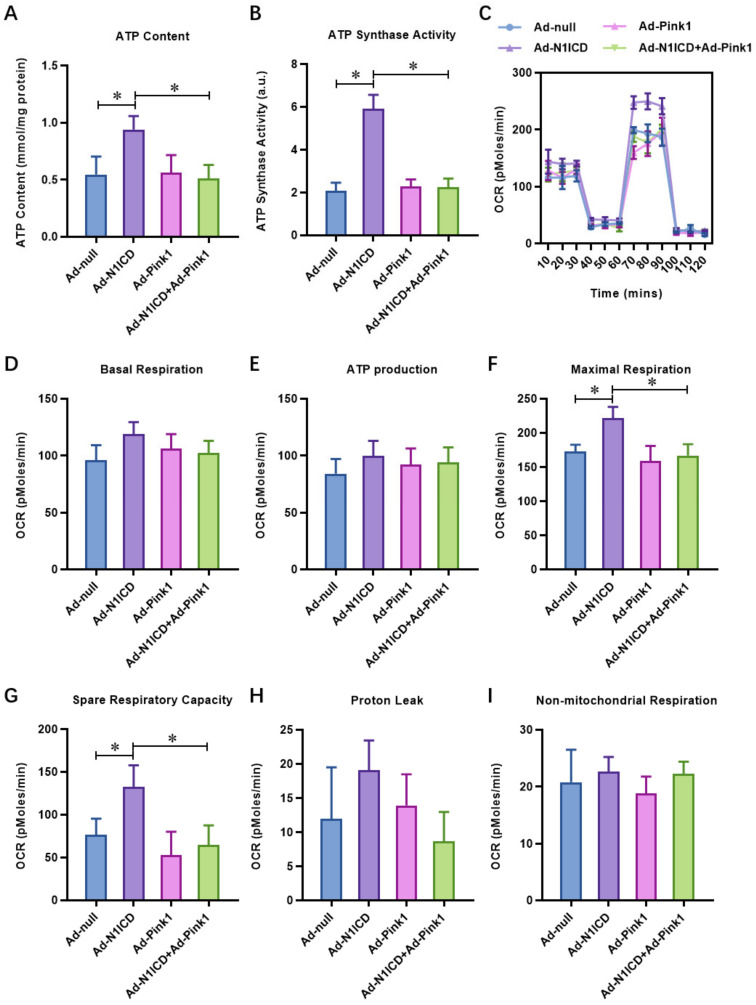
Pink1 over-expression impaired the protective effect of N1ICD against hypoxia/reoxygenation induced mitochondrial dysfunction in neonatal cardiomyocytes. (**A**) The ATP content and (**B**) ATP synthase activity was analyzed by quantitative kits; (**C**) the mitochondrial bioenergetics were measured by the seahorse XFp Extracellular Flux Analyzer. The (**D**) basal respiration (BR), (**E**) ATP production, (**F**) maximal respiration (MR), and (**G**) spare respiratory capacity (SPR), (**H**) proton leak, and (**I**) non-mitochondrial respiration were analyzed; N = 3; * *p* < 0.05 versus indicated group.

**Figure 7:**
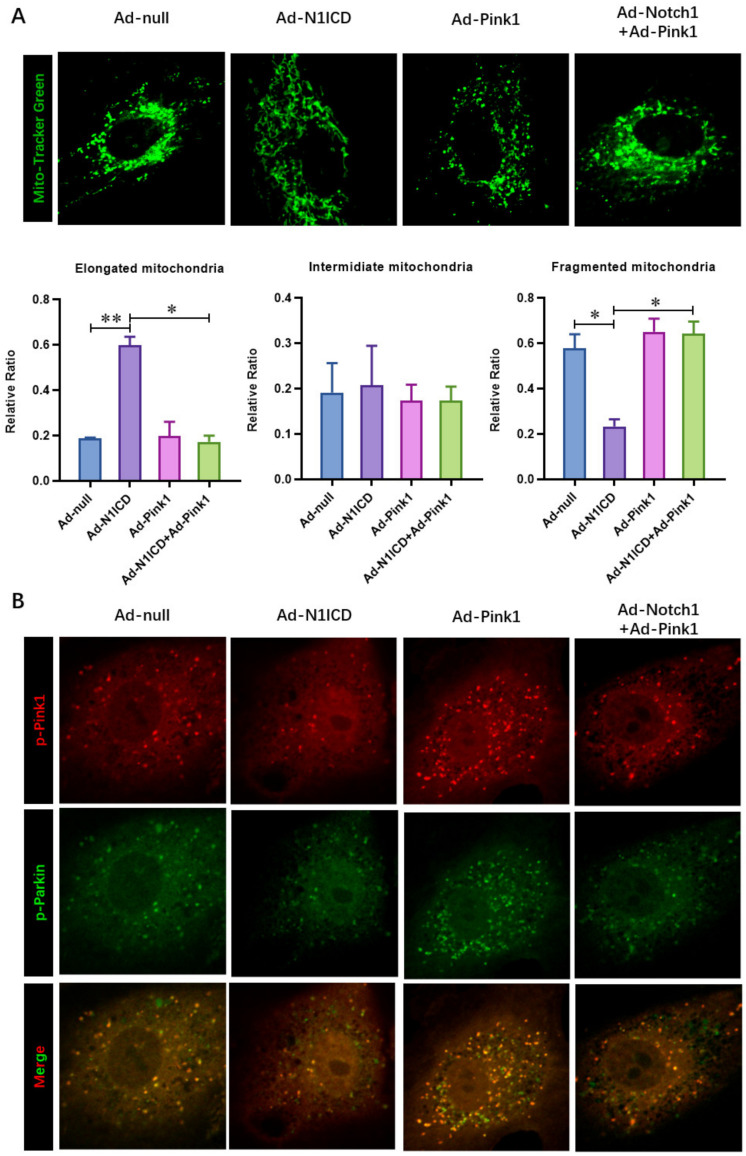
Pink1 over-expression impaired the protective effect of N1ICD against hypoxia/reoxygenation-induced mitochondrial fragmentation and mitophagy in neonatal cardiomyocytes. (**A**) Mito-tracker Green staining and confocal microscopy were used to observe the morphology of mitochondrial fragmentation in neonatal cardiomyocytes cells; (**B**) the mitophagy in cardiomyocytes was analyzed via the colocalization of GFP-LC3 puncta with Mito-TMRE-stained mitochondria as an index of mitophagy in neonatal cardiomyocytes cells; N = 3; * *p* < 0.05, ** *p* < 0.01 versus indicated group.

Similar to PTEN over-expression, we found that Notch1-preserved ATP content (Figure 6A) and ATP synthase activity (Figure 6B) in H/R neonatal cardiomyocytes were significantly decreased by Pink1 over-expression. Furthermore, the Notch1-preserved oxygen consumption rate of neonatal cardiomyocytes, including maximal respiration (MR) and spare respiratory capacity (SPR), obviously decreased with Pink1 over-expression (Figure 6C). Consistently, Notch1 decreased mitochondrial fragmentation (Figure 7A), and mitophagy (Figure 7B) was increased by Pink1 over-expression. These results demonstrate that Pink1 over-expression impairs the protective effect of N1ICD against hypoxia/reoxygenation-induced mitochondrial dysfunction and fragmentation in neonatal cardiomyocytes.

### 3.6. N1ICD Protects against Ischemic Reperfusion Injury by Suppressing PTEN-Pink1-Mediated Mitochondrial Dysfunction and Fragmentation In Vivo

**Figure 8:**
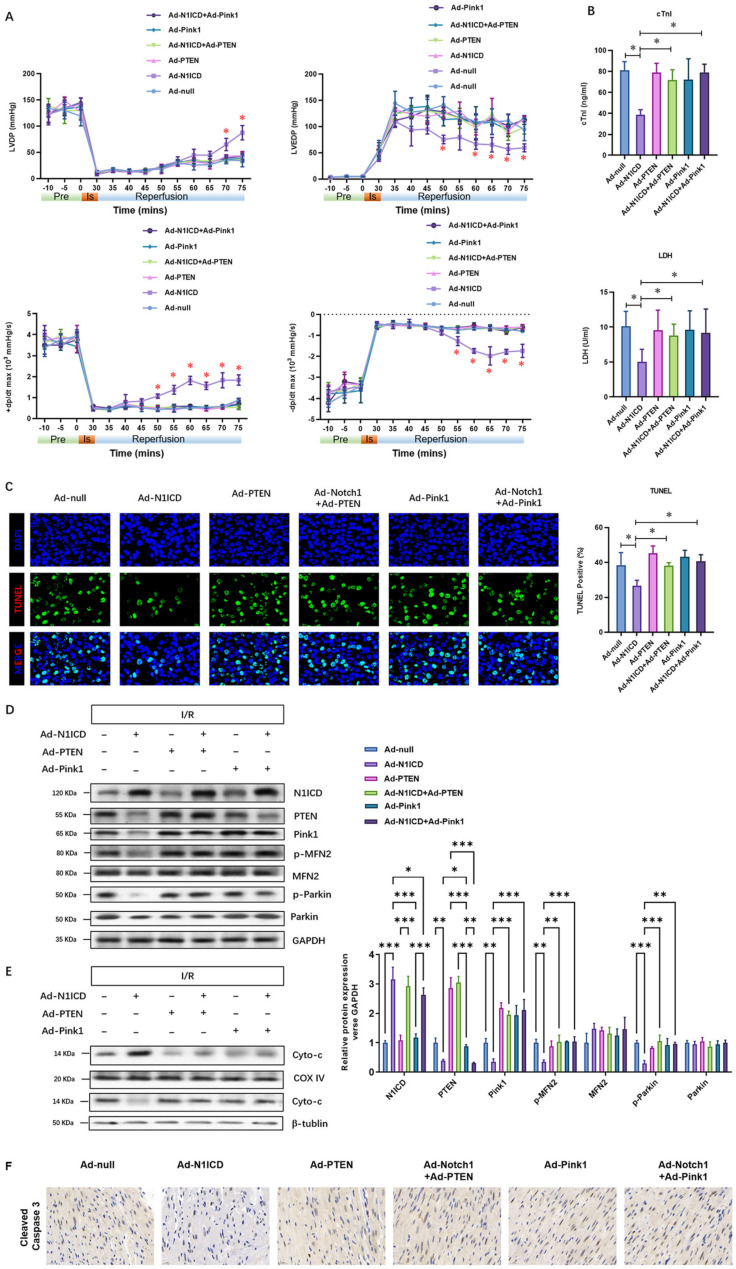
N1ICD protects against I/R injury via suppressing PTEN-Pink1-mediated mitochondrial dysfunction and mitophagy in vivo. (**A**), Representative traces and summarized data of LV pressure (LVP) during I/R in isolated rat hearts from indicated groups; (**B**) up panel: The level of cTnI was detected at the end of the reperfusion to assess myocardial injury; down panel: The activity of LDH was evaluated by electrochemiluminescence immunoassay; (**C**) representative pictures and quantitative results of TUNEL-stained cardiac sections are shown; (**D**) the regulatory molecules of mitochondrial fission and fusion, including Pink1, Parkin1, and MFN2, were analyzed; (**E**) the regulatory molecule of mitochondrial pathway apoptosis, indicated by cytochrome c release from mitochondria, was analyzed; (**F**) the expression of cleaved caspase 3 was analyzed by IHC; N = 5; * *p* < 0.05, ** *p* < 0.01, *** *p* < 0.001 versus indicated group.

To confirm the critical role of suppressed PTEN-Pink1 signaling in the cardioprotective effect of Notch1 in vivo, as shown in Figure 8A, we over-expressed Notch1 with and without PTEN/Pink1 and analyzed the post-ischemic contractile function in Langendorff-perfused rat hearts. The pre-ischemic contractile parameters were similar between the five groups, while the I/R (30 min/45 min)-suppressed LV contractile function, characterized by LV development pressure (LVDP), LV end-diastolic pressure (LVEDP), and maximal speed of LV pressure development and decline (±dp/dt), was markedly alleviated by Notch1 over-expression (Figure 8A). However, with the over-expression of PTEN or Pink1, the cardioprotective effects of Notch1 on the contractile function were totally abrogated (Figure 8A). Consistently, I/R-induced cTnI level (Figure 8B, up panel) and LDH activity (Figure 8B, down panel) in the coronary perfusate were significantly inhibited by Notch1 over-expression and dramatically impaired by the over-expression of PTEN or Pink1, respectively. Moreover, the apoptotic percentage of myocardium was determined by the TUNEL assay, and the results indicated that the positive staining of TUNEL in the Notch1 groups was significantly decreased. However, this was obviously increased in PTEN- and Pink1-over-expressed hearts (Figure 8C). Similarly, the levels of mitochondrial fragmentation markers, including Pink1, phosphorylated MFN2, and phosphorylated Parkin, were inhibited by Notch1 over-expression but reversed by PTEN over-expression (Figure 8D). Furthermore, Notch1 over-expression significantly decreased the cytoplastic cytochrome c, whereas PTEN or Pink1 over-expression elevated it (Figure 8E). Similarly, Notch1 over-expression significantly decreased the protein level of cleaved caspase 3, whereas PTEN or Pink1 over-expression elevated it (Figure 8F). Collectively, PTEN over-expression impaired the protective effect of N1ICD against hypoxia/reoxygenation-induced mitochondrial dysfunction and fragmentation in neonatal cardiomyocytes. Collectively, these results demonstrate that N1ICD protects against ischemic reperfusion injury by suppressing PTEN-Pink1-mediated mitochondrial dysfunction and fragmentation in vivo.

## 4. Discussion

Mitochondria have become important participants and regulators of myocardial ischemia-reperfusion injury [23,24,25,26,27,28]. Emerging studies have shown that Notch1 protects the heart from I/R damage, but the underlying mechanism is not fully understood. We previously reported that Notch1 activates the RISK/SAFE/HIF-1 alpha signal, reduces ROS in cardiomyocytes, enhances cardiomyocyte viability, improves mitochondrial fusion, and significantly reduces myocardial I/R injury [17,18,21,22]. In this study, we demonstrate that Notch1 preserves mitochondrial respiration and inhibits mitochondrial fragmentation by suppressing the Pink1-Parkin signaling pathway in I/R-injured neonatal cardiomyocytes and hearts.

The Notch signaling pathway is a conserved pathway guided by ligand-dependent processes that ultimately release the Notch intracellular domain (N1ICD) from its membrane progenitor cells, allowing translocation to the nucleus to initiate transcription [29,30,31,32]. In the heart, Notch is expressed in a variety of cell types, including cardiomyocytes, smooth muscle cells, and endothelial cells [12]. We previously found that the Notch1 signal is activated in the process of myocardial ischemic pre-conditions and ischemic post-conditions, improving cell viability and inhibiting apoptosis [17]. In addition, the activated Notch1 signal stabilizes mitochondrial membrane potential and reduces IRI-induced reactive oxygen species. In addition, in the Langendorff cardiac perfusion model, the activated Notch1 signal restores cardiac function, reduces lactate dehydrogenase release, and limits infarct size after myocardial ischemia [17]. We also reported that Notch1 can reduce mitochondrial lysis, reduce myocardial infarct size, and inhibit ventricular remodeling. However, the downstream target of Notch1 on the mitochondrial regulation remains unclear. Herein, we further found that N1ICD protects against I/R injury by suppressing PTEN-Pink1-mediated mitochondrial dysfunction and fragmentation.

Pink1 has a total length of 581 amino acids, including an N-terminal mitochondrial targeting sequence, a transmembrane domain, a highly conserved serine/threonine kinase domain, and a C-terminal autoregulatory domain. Under physiological conditions, due to the rapid degradation of PINK1, the level of PINK1 is low [33,34,35]. Pink1 acts upstream of Parkin and is necessary for Parkin to activate and recruit depolarized mitochondria [36,37]. Pink1 and Parkin have been shown to regulate many different mitochondria-related activities, such as biogenesis, integrity, respiration, fission, and fusion, in addition to their roles in the execution of autophagy [20,38,39,40]. In this study, we showed that Pink1 over-expression impaired the protective effect of N1ICD against hypoxia/reoxygenation-induced injury in neonatal cardiomyocytes. Moreover, Pink1 over-expression impaired the protective effect of N1ICD against hypoxia/reoxygenation-induced mitochondrial dysfunction, fragmentation, and mitophagy in neonatal cardiomyocytes. Through the in vivo data, we verified that N1ICD protects against I/R injury by suppressing PTEN-Pink1-mediated mitochondrial dysfunction, fragmentation, and mitophagy, which are critical to the mitochondrial quality control and homeostasis and essential of cellular adaptation in response to cellular metabolic state and stress.

## 5. Conclusions

This study details the mechanism by which Notch1 impairs mitochondrial dysfunction and fragmentation via the suppression of PTEN-Pink1-Parkin signaling during myocardial protection. Further studies are needed to determine the clinical significance of these effects and to develop new therapies for patients with I/R, as treating this disease remains clinically challenging.

## Data Availability

The data in this study are available from the corresponding author on reasonable request.

## Abbreviations

I/R	Ischemia/reperfusion injury;
H/R	Hypoxia/reoxygenation;
Notch	The neurogenic locus notch homolog protein;
NICD	The Notch1 intracytoplasmic domain;
cTnI	Cardiac troponin I;
LDH	Lactate dehydrogenase;
SOD	Superoxide dismutase;
CK	Creatine kinase-MB;
