# Correction: Xu et al. Notch1 Protects Against Ischemic-Reperfusion Injury by Suppressing PTEN-Pink1-Mediated Mitochondrial Dysfunction and Mitophagy. *Cells* 2023, *12*, 137

**DOI:** 10.3390/cells15030262

**Published:** 2026-01-30

**Authors:** Qirong Xu, Sheng Liu, Qiang Gong, Rongrong Zhu, Jichun Liu, Qicai Wu, Xueliang Zhou

**Affiliations:** 1 Department of Thoracic Surgery, The First Affiliated Hospital, Nanchang University, Nanchang 330006, China; ndyfy02370@ncu.edu.cn; 2 Department of Cardiac Surgery, The First Affiliated Hospital, Nanchang University, Nanchang 330006, China; ndyfy01290@ncu.edu.cn (S.L.); gongqiang@ncu.edu.cn (Q.G.); ndefy18002@ncu.edu.cn (J.L.); 3 Department of Cardiology, Jiangxi Hospital of Traditional Chinese Medicine, Jiangxi University of Chinese Medicine, Nanchang 330006, China; zhurongrong@jxutcm.edu.cn

## Error in Figure

In the original publication [1], there was a mistake in Figure 1D as published. The band size of the amplified PTEN promoter corresponding to the representative ChIP-RT-PCR gel image in this panel is incorrect. The corrected Figure 1 appears below. The authors state that the scientific conclusions are unaffected. This correction was approved by the Academic Editor. The original publication has also been updated.

## Figures and Tables

**Figure 1:**
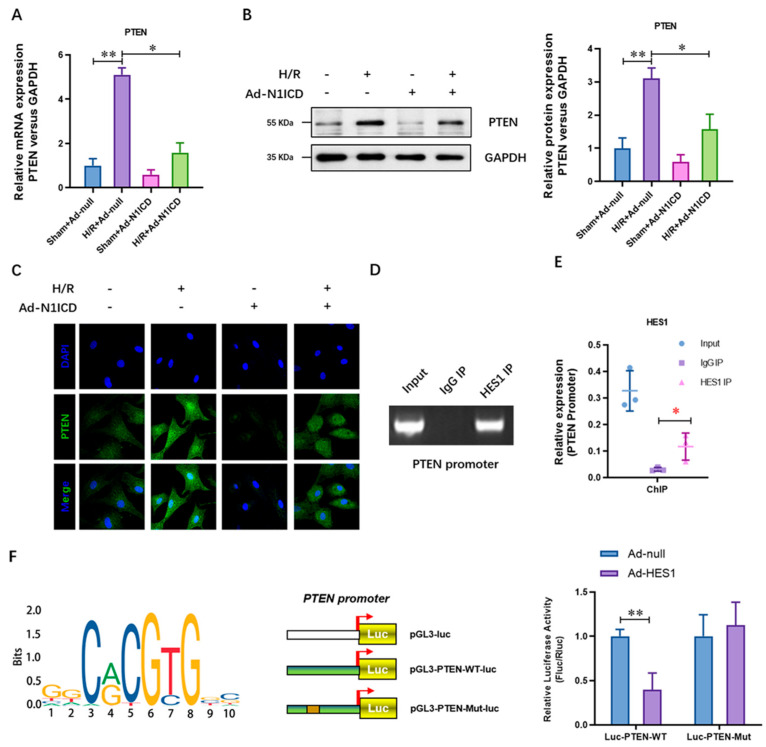
N1ICD impaired the hypoxia/reoxygenation elevated expression of PTEN in neonatal cardiomyocytes. (**A**) The mRNA level of PTEN was analyzed by real-time PCR; (**B**) the protein level of PTEN was analyzed by real-time PCR; (**C**) the protein level of PTEN was analyzed by immunofluorescence staining assay; (**D**) ChIP-RT-PCR and (**E**) ChIP-qPCR were performed to analyze the enrichment of PTEN promoter in the ChIP products of Hes1; (**F**) the promoter activity of PTEN was evaluated by luciferase assay; N = 3; * *p* < 0.05, ** *p* < 0.01 versus indicated group.
